# Editorial: Immunotherapy resistance and chronic inflammation

**DOI:** 10.3389/fimmu.2025.1737983

**Published:** 2025-11-13

**Authors:** Titto A. Augustine, Sushmita Chakraborty, Vijay Kumar

**Affiliations:** 1 Department of Neurological Surgery, Indiana University School of Medicine, Indianapolis, IN, United States; 2 Indiana University Melvin and Bren Simon Comprehensive Cancer Center, Indianapolis, IN, United States; 3 Proteinno MAB Tech Private limited, BioNEST- IIT G TIDF, IIT Guwahati, Guwahati, India; 4 Department of Surgery, Laboratory of Tumor Immunology and Immunotherapy, Morehouse School of Medicine, Atlanta, GA, United States

**Keywords:** SIRT 1 (sirtuin 1), PCOS (polycystic ovarian syndrome, COPD - Chronic obstructive pulmonary disease, CAR T cell, MIS-C (multisystem inflammatory syndrome in children), Kawasaki disease (KD), immune checkpoint inhibitor associated myocarditis (ICI-MC), LongCOVID-19

Modern immunotherapies from immune checkpoint inhibitors (ICIs) to chimeric antigen receptor (CAR)-T cell therapies and biologics have reshaped the therapeutic landscape for cancer, autoimmune disorders, and certain chronic diseases by harnessing the body’s own immune system to target disease processes. Yet, despite remarkable successes, a sizable proportion of patients either fail to respond initially or acquire resistance over time. One underappreciated but increasingly recognized obstacle is chronic, low-grade inflammation, a persistent, dysregulated immune milieu that can blunt antitumor immunity or skew immune responses away from therapeutic benefit. At the nexus of immunotherapy resistance and inflammation lies a complex interplay of molecular regulators (such as Silent Information Regulator 2 Homolog 1, aka SIRT1), immune cell phenotypes, cytokine networks, and pathological stressors that drive immune exhaustion, tolerance, and adaptation. The eight manuscripts collected - spanning mechanistic reviews, meta-analyses, and disease-specific studies - converge on a central theme: chronic inflammatory processes, sustained by molecular regulators, maladaptive immune circuits, extracellular messengers, or pathogenic antibody responses, reprogram immunity in ways that either blunt therapeutic efficacy or raise the risk of severe adverse events. Understanding these mechanistic links is essential to design combination strategies that preserve anti-disease immunity while limiting collateral inflammatory harm.

Among the key contributions, the meta-analysis of SIRT1 and inflammation argues for a nuanced role of SIRT1 as both a sensor and modulator of inflammatory states, with levels apparently rising in many inflammatory conditions yet varying by age, disease type, and sample modality. In parallel, disease-oriented studies traverse a broad disease spectrum polycystic ovary syndrome (PCOS) delineates the contribution of systemic low-grade inflammation to chronic metabolic and reproductive dysregulation ; post-acute sequelae of SARS-CoV-2 implicate extracellular vesicle–mediated inflammatory signaling as a persistent inflammatory stimulus; and non-eosinophilic chronic obstructive pulmonary disease (COPD) emphasizes how type 1 immune signatures (e.g. CXCL9) underlie a subtype of airway inflammation resistant to conventional therapies. In the immunotherapy domain, the retrospective comparison of CD19/20 dual-targeted CAR-T in relapsed/refractory DLBCL indicates how even in precision immunotherapy settings, expansion, persistence, and toxicity are modulated by underlying immune contexture. Collectively, these works underscore that chronic inflammation is not a passive backdrop, but an active forest of resistance mechanisms ranging from regulatory feedback loops to skewed immune polarization that must be understood and overcome for durable immunotherapeutic success.

Chronic, dysregulated inflammation is increasingly recognized as an active driver of therapeutic failure rather than a passive backdrop. The Research Topic opens with a systematic review and meta-analysis on SIRT1, a stress-responsive deacetylase that intersects NF-κB and other inflammatory circuits, underscoring SIRT1’s tight linkage to inflammatory states and its potential as a biomarker or target to rebalance maladaptive immune tone. The manuscript by Sun et al. comprehensively examines how central metabolic sensors and deacetylases influence cytokine networks and oxidative stress. SIRT1’s role in deacetylating transcription factors (e.g., NF-κB) and modulating redox balance positions it as a nodal controller of inflammatory tone. In therapeutic contexts, SIRT1 dysregulation can sustain pro-inflammatory cytokines, such as TNF-α, IL-6, and C-reactive protein (CRP), production and immune-suppressive microenvironments (for example, via chronic NF-κB activation), undermining the ability of therapies that rely on reinvigorating anti-tumor or antiviral T cells. Practically, this suggests metabolic or epigenetic modulation (restoring SIRT1 activity) as a complementary approach to improve immunotherapy responsiveness.

This systems lens is then grounded in disease contexts where persistent inflammation remodels tissues and immune set-points: PCOS is framed as a condition of systemic low-grade inflammation with intrinsic endocrine–immune crosstalk, while multisystem inflammatory syndrome in children (MIS-C) and Kawasaki disease (KD) are contrasted as pediatric hyperinflammatory syndromes with overlapping vascular and cytokine signatures. Deng et al. studied the role of chronic low-grade inflammation as a central contributor to the pathogenesis of PCOS. The study highlights that women with PCOS exhibit elevated inflammatory markers, including CRP, TNF-α, and IL-6, which are strongly associated with insulin resistance, oxidative stress, and hormonal imbalance. It further explores how intrinsic mechanisms, such as, adipose tissue dysfunction, hyperandrogenism, and gut microbiota alterations sustain this inflammatory state. The paper also recommends that a potential diagnostic test panel could include markers that reflect the inflammatory state and metabolic health of PCOS patients. Overall, the paper emphasizes inflammation as both a cause and consequence of PCOS, suggesting that targeting inflammatory pathways could offer new therapeutic strategies for managing the condition.

On the adverse-event side, a case series combined with a systematic review of immune checkpoint inhibitor–associated myocarditis (ICI-M) reports potential advantages of initial high-dose methylprednisolone pulses, such as faster declines in cardiac injury biomarkers and lower post-treatment MACE and cardiovascular mortality, while also cautioning about the oncologic trade-offs of aggressive immunosuppression—highlighting the delicate balance between controlling harmful inflammation and preserving antitumor immunity. Moreover, Man et al. investigates optimal steroid dosing strategies for managing ICIs-induced myocarditis. Through a combination of case series and systematic review, the authors compared clinical outcomes between high-dose and low-dose corticosteroid treatments. Several advantages of high-dose therapy were identified, including faster and more stable declines in cardiac injury biomarkers (cTnI/T, CK, NT-proBNP), quicker symptom resolution with a simpler treatment course, and lower rates of MACE and cardiovascular mortality compared with low-dose therapy. However, since the study was based on a small case series and retrospective analysis, these findings should be interpreted with caution. The research emphasizes the need for individualized treatment approaches and calls for larger, controlled trials to establish evidence-based guidelines for steroid use in ICI-M.

In another study, Lupu et al. explores the clinical, immunological, and pathological similarities and differences between MIS-C and KD. The authors’ review emerging evidence showing that both conditions share overlapping inflammatory pathways, including cytokine dysregulation and endothelial injury, yet differ in their triggers and immunophenotypes. MIS-C, often following SARS-CoV-2 infection, tends to present with more severe cardiac involvement and systemic inflammation compared to classical KD. The study underscores the importance of distinguishing between the two for accurate diagnosis and tailored treatment, while also suggesting that insights from MIS-C research could enhance understanding of the broader spectrum of pediatric inflammatory diseases.

Furthermore, Long-COVID is another chronic inflammatory manifestation of COVID-19. Bachiller et al. have extended Long-COVID-associated inflammatory events to extracellular vesicles (EVs). According to the group, fifteen months after infection, EV cargo and inflammatory mediators remain perturbed, supporting a mechanism by which sustained, vesicle-borne signaling helps perpetuate post-acute sequelae. This publication investigates how EVs contribute to the persistent inflammation observed in post-acute sequelae of SARS-CoV-2 infection (PASC), commonly referred to as long COVID. Bachiller et al. identifies that EVs derived from infected individuals carry pro-inflammatory molecules and viral components that can activate immune and endothelial cells, perpetuating systemic inflammation even after viral clearance. These findings suggest that EV-mediated signaling plays a key role in sustaining immune dysregulation and tissue damage associated with long-term COVID-19 symptoms. The authors propose that targeting EV pathways could represent a novel therapeutic strategy for mitigating chronic inflammation in PASC. EV-mediated delivery of cytokines, viral antigens, or RNA fragments can chronically stimulate innate and adaptive cells, maintaining a pro-inflammatory milieu. In oncology or autoimmunity, analogous EV signaling could perpetuate immunosuppressive myeloid states or T-cell dysfunction, contributing to resistance to ICIs or to relapses after adoptive cell therapies.

In respiratory disease, a non-eosinophilic COPD subtype defined by CXCL9 and type-1–skewed immunity illustrates how heterogeneity inside “chronic inflammation” yields distinct immune-therapy implications and biomarker strategies. Chengsheng et al. identifies a distinct non-eosinophilic subtype of COPD characterized by elevated CXCL9 levels and activation of type 1 immune pathways. Through transcriptomic and immunological analyses, the study reveals that this subtype is driven by interferon-γ–mediated inflammation rather than eosinophilic or type 2 immune mechanisms. Patients exhibiting this profile showed more severe airway inflammation and poorer responses to corticosteroid therapy. The findings highlight CXCL9 as a potential biomarker for stratifying COPD patients and suggest that targeting type 1 immune signaling could provide new therapeutic avenues for managing non-eosinophilic COPD. Translating this insight, when immunotherapy resistance stems from type-1–skewed inflammation, standard anti-inflammatory strategies (like steroids) may be ineffective or deleterious. The myocarditis study comparing high- versus low-dose steroids in ICI-induced myocarditis (ICI-M) reinforces the clinical tension: high-dose steroids can blunt dangerous inflammation but risk undermining the protective immune responses, aggravating the treatment-associated complications. Thus, precise phenotyping of inflammatory subtypes (e.g., CXCL9-high/type-1) is critical for choosing immunomodulatory strategies that do not negate therapeutic efficacy.

Clinical findings by Xue et al. translates these insights to immunotherapy outcomes and safety. In hematologic oncology, dual-target CD19/20 CAR-T cells achieved markedly higher early objective and complete response rates than single-target CD19 CAR-T in relapsed/refractory DLBCL-yet the work also hints that expansion, persistence, and toxicity remain coupled to the host inflammatory milieu, reinforcing the need to co-manage inflammatory context with cell therapy. The study evaluates the clinical outcomes of dual-target CD19/20 CAR-T-cell therapy compared with traditional CD19-targeted CAR-T therapy in patients with relapsed or refractory diffuse large B-cell lymphoma (R/R DLBCL). It found that CD19/20 dual-target CAR-T therapy achieved higher overall and complete response rates while reducing relapse linked to antigen loss. Although both treatments showed comparable safety profiles, dual-target therapy was associated with slightly lower rates of severe cytokine release syndrome and neurotoxicity. The inflammatory microenvironment influenced CAR-T persistence, exhaustion, and toxicity. A chronic inflammatory milieu enriched in specific cytokines or suppressive myeloid cells could accelerate CAR-T dysfunction or increase severe toxicity. Dual targeting is one approach to antigen escape, but tackling the inflammatory environment (e.g., modulating myeloid cells, EV signaling, or metabolic regulators) is necessary to enhance durability and safety. These findings suggest that CD19/20 CAR-T therapy may provide a more effective and durable treatment option for R/R DLBCL patients.

Finally, work on cryoglobulinemic glomerulonephritis (CryoGN), a rare but severe renal complication in patients with cryoglobulinemia, evaluates the renal prognostic value of serum monoclonal immunoglobulin (mIg), pointing to how immune-complex biology and intrarenal immune cell patterns may refine risk and guide intervention in inflammation-driven renal injury(8). Through retrospective analysis, Ma et al. found that the presence of mIgs was associated with more severe renal impairment, higher proteinuria levels, and poorer long-term renal prognosis compared to patients without monoclonal components. The findings suggest that serum mIg serves as a key prognostic biomarker, helping to identify CryoGN patients at greater risk of progression to chronic kidney disease. The authors highlight the importance of early detection and individualized management strategies to improve renal outcomes in this patient population. In the immunotherapy era, antibody (Ab)-mediated pathology (either preexisting or therapy-induced) can complicate outcomes and limit tolerable dosing. Recognizing antibody-driven inflammatory axes is therefore essential when planning or monitoring immune interventions.

Taken together, these studies illuminate a multi-layered model by which chronic inflammation undermines immunotherapy: (1) upstream regulators (SIRT1, metabolic shifts) establish a permissive pro-inflammatory state; (2) intercellular mediators (EVs, chemokines like CXCL9) propagate and localize that inflammation; (3) immune polarization (type-1 vs type-2 vs eosinophilic) dictates responsiveness to standard immunomodulators; and (4) antigenic/autoantibody factors determine structural targets and long-term organ outcomes. To overcome resistance, precision immunotherapy must therefore incorporate inflammatory phenotyping - molecular (SIRT1, interferon signatures), cellular (myeloid vs lymphoid composition), and extracellular (EV cargo, chemokine profiles) - and apply rational combination strategies: metabolic/epigenetic modulators, targeted anti-inflammatory agents (rather than broad-spectrum high-dose steroids), EV pathway inhibitors, and combinatorial antigen targeting for cell therapies.

This strategy reframes “resistance” as a dynamic mismatch between therapy and host inflammatory programs and suggests that aligning the two can convert transient responses into durable control while minimizing immune-related harm. Clinically, these manuscripts argue for (a) routine assessment of inflammatory biomarkers before and during immunotherapy, (b) adaptive dosing and selective immunosuppression when needed (to control life-threatening inflammation without abolishing anti-disease immunity), and (c) translational trials that pair immunotherapies with agents that correct the specific inflammatory drivers identified in patients (e.g., SIRT1 agonists/modulators, CXCL9/IFN-γ pathway inhibitors, or EV-targeted approaches). Such integrated approaches offer the best path to durable responses while minimizing toxicity in diverse patient populations affected by chronic inflammation.

